# Peripheral blood-derived mesenchymal stem cells in osteochondral repair: a perspective on emerging insights and future directions

**DOI:** 10.3389/fbioe.2026.1734349

**Published:** 2026-03-26

**Authors:** Senthilkumar Rajendran, Pier Paolo Parnigotto, Antara Banerjee, Silvia Barbon

**Affiliations:** 1 Foundation for Biology and Regenerative Medicine, Tissue Engineering and Signaling-T.E.S. ETS, Padova, Italy; 2 Faculty of Allied Health Sciences, Chettinad Academy of Research and Education (CARE), Chettinad Hospital and Research Institute (CHRI), Chennai, India; 3 Department of Neuroscience, Section of Human Anatomy, University of Padua, Padua, Italy

**Keywords:** autologous therapies, immunotherapy, multipotent stem cells, osteochondral damage, peripheral blood

## Abstract

Mesenchymal stem cells (MSCs) isolated from peripheral blood (PB) are gaining increasing attention among both researchers and clinicians, representing a promising alternative to traditional stem cell sources such as bone marrow and adipose tissue. Their therapeutic use offers key advantages like high accessibility, minimally invasive collection and autologous nature of the graft. Together with the ability to differentiate into multiple cell types, PB-MSC features make them ideal candidates for a wide range of clinical applications in the fields of regenerative medicine, tissue engineering and immunotherapy. While the therapeutic potential of PB-derived MSCs is more and more acknowledged, several challenges still need to be overcome regarding, for example, their isolation, expansion, and differentiation efficiency. For clinical translation, PB-MSC administration may be specifically indicated when minimally invasive autologous cell therapies are required, especially when bone marrow harvest is not recommended due to age, comorbidity, or prior surgeries. Conversely, PB-MSC auto-transplant should be avoided in patients suffering from hematological malignancies, active infections, or bone marrow disorders, where circulating stem cell populations may be altered, or their isolation poses safety risks. In this Perspective, we highlight recent advances in PB-MSC isolation and characterization, discuss their bioengineering integration into osteochondral repair strategies, and examine their immunomodulatory potential in osteoarthritis (OA). We propose that PB-MSCs integrate regenerative and immunomodulatory properties, positioning them as a promising, autologous cell source for next-generation personalized regenerative therapies. However, their clinical translation critically depends on the development of reproducible, GMP-compliant expansion protocols and validated potency assays.

## 1 Introduction

Mesenchymal stem cells (MSCs) isolated from peripheral blood (PB) are emerging as an innovative resource in regenerative medicine, tissue engineering and immunotherapy, representing a promising option to replace traditional sources like bone marrow, adipose tissue, or embryonic tissues. One of the most important advantages offered by the use of PB-MSCs is their high accessibility and the minimally invasive nature of their isolation procedure. Indeed, bone marrow aspiration could be painful and pose certain risks for the donor, whereas peripheral blood collection is easy, safe, and not harmful for patients. This prompts a wider range of PB-MSC applications in translational medicine, making them a compelling cell source for both research and therapeutic use (Barbon et al., 2021; Di Liddo et al., 2016). As further advantage, MSC isolation from adult PB does not raise the ethical issues which are commonly related to the use of embryonic tissues, allowing for larger acceptance and application of stem cell-based therapies. In addition, the renewable nature of PB must also be taken into consideration, since it enables repeated sample collection for consistent amount of stem cells retrieval, without causing significant harm or discomfort to donors. No less importantly, PB is a stem cell source which can be used for autologous therapies, highly reducing the risk of immune rejection or any complications associated with allogenic transplants (Chen et al., 2020; Li et al., 2015).

This perspective article presents recent and relevant studies focusing on the preclinical and clinical use of PB-MSCs for osteochondral repair, providing insights into current advancements, existing challenges, and the translational potential of their clinical application. To this end, a targeted literature search using Scopus, and Web of Science was performed, with the following keywords: (“Peripheral Blood-Derived Mesenchymal Stem Cells” OR “PB-MSC” OR “peripheral blood stem cells”) AND (“osteochondral repair” OR “cartilage regeneration” OR “osteoarthritis” OR “chondral lesion”). Included studies focused on isolation, characterization, preclinical applications, immunomodulation, or clinical translation of PB-MSCs in joint repair.

## 2 Isolation and characterization of peripheral blood-derived MSCs

The development of standardized protocols for PB-MSC isolation and characterization play a pivotal role to support their effective use in both research and clinical applications. Unlike bone marrow-derived MSCs, PB-MSCs circulate in the bloodstream at low frequencies, which makes their isolation particularly challenging and causes variability in cell yield, purity, and functional properties (Churchman et al., 2020). Standardizing PB-MSC isolation would allow to obtain more consistent outcomes in cell viability, specific immunophenotype, and multi-differentiation potential, supporting the possibility for their translational application. Specifically, donor-related variability, cell mobilization techniques, sorting strategies (e.g., Ficoll gradient centrifugation, flow cytometry), and culture conditions are among the key factors that need to be refined to improve PB-MSC extraction efficiency. Besides that, standardizing characterization protocols is equally crucial, since PB-MSCs must be reliably identified from other circulating stem/progenitor cells (i.e., hematopoietic stem cells, endothelial progenitor cells). Immunophenotype characterization should be based on the identification of a defined set of cell surface markers (e.g., CD29, CD73, CD90, and CD105), while ensuring the negative expression of hematopoietic targets like CD34 and CD45 (Lin et al., 2019; Dominici et al., 2006). Additionally, investigating PB-MSC differentiation potential into three or even more different cell lineages (e.g., osteocytes, chondrocytes, and adipocytes), as well as their immunomodulatory properties, appear to be fundamental to confirm stem cell identity, functionality and therapeutic relevance.

Recent research highlighted that PB-MSC isolation can rely on efficient protocols mainly based on blood sample enrichment using Ficoll density gradient centrifugation followed by flow cytometry cell-sorting to select specific MSC markers. Isolated PB-MSCs showed to retain multi-differentiation potential responding to adipogenic, chondrogenic and osteogenic induction (Mattei et al., 2025; Lin et al., 2019).

Along with laboratory-developed protocols, different commercial products have been made available to extract MSCs from peripheral blood, addressing the specific demand for standardized, reproducible, and scalable isolation methods. These products vary from density gradient media and immunomagnetic selection kits for research use only, to automated closed-system devices applicable to Good Manufacturing Practice (GMP)-compliant clinical procedures (Table 1). The commercial availability of these products is particularly relevant for translational applications, when reducing inter-operator variability, improving cell yield and ensuring regulatory compliance are key operational objectives. Nevertheless, the supply of GMP-compliant products for clinical-grade PB-MSC isolation still appear to be limited, highlighting a procedural gap that needs to be addressed in the near future.

**TABLE 1:** Commercial products for PB-MSC isolation.

Product name	Manufacturer	Principle/Method	Target cell type	Regulatory status
RosetteSep™ MSC enrichment cocktail	STEMCELL technologies	Immunodensity-based negative selection	PB-MSCs BM-MSCs	Research use only
EasySep™ human MSC enrichment kit	STEMCELL technologies	Immunomagnetic positive/negative selection	CD105^+^/CD73^+^/CD90^+^ MSCs	Research use only
Ficoll-Paque™	Cytiva	Density gradient centrifugation	MNCs (including MSCs) from PB	Research/IVD
Sepax™ cell Processing system	BioSafe (now part of Cytiva)	Automated density gradient centrifugation	MNCs from PB or apheresis	GMP-compliant (medical device)
CliniMACS® plus	Miltenyi Biotec	Immunomagnetic selection	CD271^+^ MSCs (from apheresis)	GMP-compliant (medical device)

Abbreviations: BM-MSCs, Bone Marrow Mesenchymal Stem Cells; GMP, good manufacturing practice; IVD, in vitro diagnostic; MNCs, Mononuclear Cells; MSCs, Mesenchymal Stem Cells; PB-MSCs, Peripheral Blood Mesenchymal Stem Cells.

Notably, scientific evidence suggests that the use of apheresis blood products for PB-MSC isolation may provide several advantages which may favor their clinical application. One crucial benefit is the significantly higher cell yield in comparison with standard peripheral blood collection. Apheresis enables the selective enrichment of mononuclear cells, including MSC precursors, thus improving the efficiency of PB-MSC isolation. This approach can avoid issues related to the typically low frequency of MSCs in the peripheral circulation, whereas their multipotent differentiation potential is retained, including their differentiation capacity towards osteogenic, chondrogenic, adipogenic and neurogenic lineages, similar to bone marrow-derived MSCs [3-5] (Deka et al., 2024; Jain et al., 2020; Barbon et al., 2018; Caloprisco et al., 2010).

Despite these proven advantages, further protocol standardization and optimization of expansion conditions are required to fully harness the therapeutic potential of PB-MSCs derived from apheresis products. From our perspective, the success of these approaches will depend on the development of reproducible protocols that ensure not only cell yield and purity but also functional consistency across batches, which is an essential step toward enabling their clinical scalability.

### 2.1 Isolation of peripheral blood- *versus* bone marrow- and adipose tissue-MSCs

A critical evaluation of PB-MSC translational potential into the clinical setting requires comparison with the more established bone marrow-derived MSCs (BM-MSCs) and Adipose-derived MSCs (AD-MSCs), particularly in terms of cell source accessibility, cellular yield, and isolation efficiency, with the aim of contextualizing their emerging role in regenerative applications.

Both PB-MSCs, BM-MSCs and AD-MSCs offer distinct advantages and limitations in terms of isolation strategy (Table 2). PB-MSCs are accessible through a simple blood collection, making them minimally invasive and highly favorable for repeat sampling and autologous therapies. However, they circulate in peripheral blood at low frequency under normal conditions, requiring enrichment techniques such as Ficoll density gradient centrifugation, immunoselection, mobilization or apheresis to isolate viable MSC populations. In contrast, BM-MSCs are readily harvested at higher yields and are extensively characterized for clinical use, but the invasive nature of bone marrow aspiration poses significant concern and risk, limiting donor availability and repeat procedures (Li et al., 2023).

**TABLE 2:** Isolation of PB-MSCs *versus* BM-MSCs and AD-MSCs.

Feature	PB-MSCs	BM-MSCs	AD-MSCs
Collection method	Minimally invasive (venipuncture)	Invasive (bone marrow aspiration)	Minimally invasive (liposuction/lipectomy)
Donor discomfort and risk	Very low	Moderate to high (pain, infection, bleeding)	Low to moderate (bruising, swelling at harvest site)
Autologous application feasibility	High	High	High
Sampling frequency	Repeatable	Limited	Limited (repeat harvest possible but invasive)
MSC yield (Baseline)	Very low (≤0.001% of MNCs)	High (0.001%–0.01% of MNCs)	Very high (up to 2% of nucleated cells)
Enrichment Requirements	Ficoll Flow cytometry Apheresis	Usually not necessary for initial isolation	Minimal; high yield from stromal vascular fraction
Donor variability	High	Moderate	Moderate
Phenotypic stability in culture	Less established	Well characterized	Well characterized
Regulatory acceptance	Limited clinical data	Extensive preclinical and clinical data	Growing clinical data (especially in orthopedics)
Ethical/Immunological concerns	None when autologous	Risk of rejection in allogeneic use	None when autologous

Adipose-derived MSCs may be considered as an intermediate option: they are collected through minimally invasive procedures (i.e., liposuction or lipectomy), which assure for high harvest yield of stromal cells (up to 2% of nucleated cells), thus reducing the need for *ex vivo* expansion. However, unlike PB-MSC collection, AD-MSC harvest is less repeatable since it could lead to relevant donor-site complications related to the liposuction technique, including bruising, swelling, haematoma formation, paraesthesia or donor-site pain, infection and hypertrophic scarring (Simonacci et al., 2019).

Overall, while BM-MSCs remain the reference standard due to their high cellular yield and extensively characterized biology, PB-MSCs and AD-MSCs are increasingly recognized as a valuable alternative. In our opinion, their minimally invasive collection method and suitability for autologous use confer significant logistical and ethical advantages to PB-MSCs, particularly in aging or comorbid populations where bone marrow harvest may be contraindicated. On the other hand, AD-MSCs may offer a compelling equilibrium between yield and invasiveness, resulting particularly advantageous when the therapy requires higher cell numbers with moderate discomfort at the donor site.

However, one of the most important practical limits remains the intrinsically low frequency of PB-MSCs in the peripheral circulation. Under steady-state conditions, PB-MSCs represent a very rare cell population, with estimated frequency ≤0.001% of mononuclear cells (MNCs) (Kassis et al., 2006), which is substantially lower than the 0.01%–0.1% frequency typically detected in bone marrow aspirates (Pittenger et al., 1999; Dominici et al., 2006). In order to enhance harvest yields, clinical protocols often rely on mobilization strategies such as granulocyte colony-stimulating factor (G-CSF) administration before blood collection. This procedure was proved to be effective in increasing circulating MSC numbers but showed to induce transient side effects including bone pain, fatigue, and headache (Cai et al., 2024). Furthermore, even after mobilization, PB-MSC isolation often requires additional enrichment procedures (i.e., density gradient centrifugation, immunomagnetic selection, or large-volume apheresis) to obtain clinically relevant cell numbers. These technical demands highlight the need for refined isolation protocols that balance yield, safety, and patient comfort.

Although PB-MSCs have to face limitations related to low frequency and variability, progressive improvements in isolation techniques are significantly enhancing their clinical applicability. However, to fully realize their therapeutic potential, it is essential to develop standardized isolation workflows, GMP-compliant expansion protocols, and validated potency assays that ensure consistency, safety, and efficacy across clinical use.

## 3 Applications in osteochondral regeneration

In the fields of regenerative medicine and tissue engineering, PB-derived MSCs are laying the foundation for novel treatments at the forefront of medical technology. Their ability to differentiate into different tissue-specific cell types makes them valuable candidates for repairing damaged tissues. One of the most intriguing applications of PB-MSCs is related to the repair of osteochondral defects, being these cells under investigation for their potential to regenerate cartilage injuries and address osteoarthritis (OA) pathophysiology (Zhu and Fu, 2022). Osteoarthritis hallmarks include articular cartilage (AC) deterioration and chronic inflammation in the joint, which cause debilitating painful condition and severely impair patients’ mobility and quality of life. Due to its avascular nature and low cell density, AC shows limited regenerative and self-repair capacity (Salman et al., 2023; Knights et al., 2023). Traditional therapies (e.g., autografts, microfractures, and allografts) still exhibit significant limitations, such as shortage of donor tissue and inadequate hyaline cartilage neo-formation (Armiento et al., 2019). Consequently, these approaches can only alleviate the symptoms but not address the causes of cartilage degradation. For these reasons, there is growing interest towards regenerative medicine therapies involving stem cell-based approaches, as well as tissue engineering strategies coupling stem cells with smart scaffolds (Barbon et al., 2025). From our perspective, these strategies can be recognized as promising solutions to stimulate damaged cartilage regeneration and provide more effective, minimally invasive alternatives to traditional OA treatments.

**FIGURE 1:**
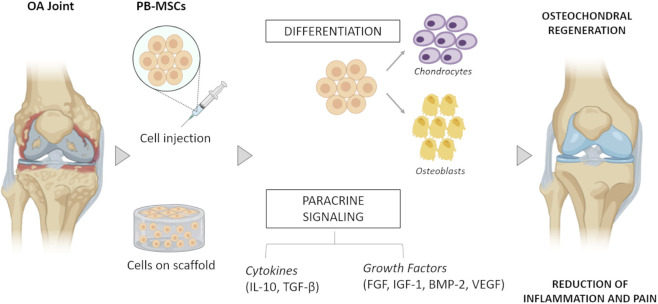
Schematic representation of PB-MSC-based regenerative strategies for osteoarthritic joints. The osteoarthritic (OA) joint environment is characterized by damaged articular cartilage, eroded subchondral bone, and synovial inflammation driven by a pro-inflammatory cytokine milieu. Therapeutic intervention involves the injection or implantation of peripheral blood-derived mesenchymal stem cells (PB-MSCs), either alone or embedded within scaffolds. PB-MSCs contribute to joint repair through two primary mechanisms: (1) differentiation into chondrocytes and osteoblasts, supporting cartilage matrix synthesis (collagen type II, aggrecan) and subchondral bone remodeling, respectively; and (2) paracrine signaling, including the secretion of anti-inflammatory cytokines (e.g., IL-10, TGF-β) and trophic/angiogenic factors (e.g., FGF, BMP-2, VEGF). These actions collectively promote regeneration of hyaline-like cartilage, restoration of subchondral bone architecture, and reduction of inflammation and pain. This figure was created using *BioRender.com*.

Within this framework, PB-MSCs emerge as suitable candidates to be integrated into such tissue engineering approaches, owing to their ease of collection, immunomodulatory properties, and capacity to differentiate into multiple tissue specific lineages. Indeed, the therapeutic efficacy of PB-MSCs is mainly ascribable to their ability to differentiate towards the chondrogenic lineage to acquire chondrocyte-like features, triggering cartilage neo-formation and joint integrity restoration, as well as counteracting the degenerative mechanisms associated with OA (Zhang et al., 2023; Wang et al., 2024) (Figure 1). While PB-MSCs possess inherent chondrogenic potential, it is important to consider that MSC-derived cartilage often resembles fibrocartilage rather than hyaline cartilage, which may limit long-term functional integration (Nejadnik et al., 2020). To address this, recent strategies focus on combining PB-MSCs with chondro-inductive biomaterials (e.g., collagen type II-enriched scaffolds) (Duan et al., 2022), growth factors (e.g., TGF-β3, BMP-2) (Lee et al., 2021), or mechanical stimulation (Chen et al., 2022), aiming to promote a more hyaline-like phenotype. Such combinatorial approaches aim not only to enhance matrix deposition but also to improve the biomechanical and biochemical properties of the regenerated tissue, bringing MSC-derived matrix closer to hyaline cartilage properties.

Supporting the therapeutic potential of PB-MSCs, multiple preclinical studies have demonstrated the regenerative capacity of PB-MSCs in promoting articular cartilage and osteochondral repair across different animal models of cartilage damage, further validating their translational applicability (Table 3).

**TABLE 3:** Overview of the preclinical and translational studies using PB-MSCs for osteochondral repair.

References	Species	Disease/Defect type	PB-MSC source and delivery	Adjunct/Scaffold	Main outcomes
Fu et al. (2014)	Rabbit	Full-thickness AC defect	Mobilized PB-MSCs, implanted	DBM	- Cartilage-like tissue formation - Chondrogenesis comparable to BM-MSCs
Zhao et al. (2018)	Pig	AC defect	Mobilized PB-MSCs, implanted	DBM added with BMP-2 and TGF-β3	Improved defect filling, cartilage organization, and integration
Lin et al. (2022)	Mouse	Post-traumatic OA	Intra-articular PB-MSC injection	-	- Reduced cartilage degeneration - Decreased catabolic/inflammatory markers
Zhang et al. (2023)	Rabbit	Surgery-induced OA	Intra-articular PB-MSCs	PRP	- Enhanced cartilage thickness and integrity - Reduced synovial inflammation
Brondeel et al. (2023)	Dog	Naturally occurring OA	Intravenous equine PB-MSCs	-	- Pain reduction and improved mobility - No major adverse reactions to the cell xenograft
Wang et al. (2024)	Mouse	Osteochondral defect	PB-MSCs, implanted	Alginate–gelatin scaffold + ICA + SDF-1α	- Upregulated expression of SOX9, collagen II, aggrecan - Significant cartilage regeneration
Mao et al. (2025)	Rabbit	AC defect	PB-MSCs loaded on hydrogel	PHBVHHx-PEG + HA + KGN hydrogel scaffold	Improved matrix deposition and defect repair
Su et al. (2025)	Rabbit	AC defect	PB-MSCs loaded on hydrogel	PHBVHHx-PEG/collagen hydrogel scaffold	- Enhanced cartilage repair - ECM synthesis - Inflammatory modulation

Abbreviations: AC, articular cartilage; BM-MSCs, Bone Marrow Mesenchymal Stem Cells; BMP-2, bone morphogenetic protein-2; DBM, demineralized bone matrix; ECM, extracellular matrix; HA, hyaluronic acid; ICA, icariin; KGN, kartogenin; OA, osteoarthritis; PB-MSCs, Peripheral Blood Mesenchymal Stem Cells; PEG, polyethylene glycol; PHBVHHx, poly (3-hydroxybutyrate-co-3-hydroxyvalerate-co-3-hydroxyhexanoate; PRP, Platelet-rich plasma; SDF-1α, stromal cell-derived factor-1α; TGF-β3, transforming growth factor-β3.

Fu and collaborators (2014) provided one of the earliest demonstrations of the applicability of PB-MSCs for cartilage repair. In their study, mobilized PB-MSCs were combined with a demineralized bone matrix (DBM) scaffold and implanted into rabbits with full-thickness cartilage lesions, exhibiting biological and chondrogenic properties comparable to bone-marrow-derived MSCs. Following implantation, PB-MSCs supported *in vivo* chondrogenesis and contributed to the formation of cartilage-like repair tissue, indicating that peripheral blood represents a viable and minimally invasive MSC source for cartilage repair strategies. In our view, this work can be regarded as a cornerstone in the field, as it directly challenged the long-standing paradigm that bone marrow represents the gold standard MSC source for cartilage regeneration.

Taking advantage of scaffold-based delivery strategies, Wang and colleagues (2024) described the local administration of PB-MSCs to the site of osteochondral damage in OA murine models by the use of alginate-gelatin scaffolds loaded with Icariin (ICA) and stromal cell-derived factor-1 alpha (SDF-1α), named as GAIS scaffolds. Alginate-gelatin hydrogels could provide mechanical support for PB-MSCs, as well as mimic the cartilage extracellular matrix (ECM) to promote cell adhesion, proliferation, and differentiation. In parallel, the addition of ICA and SDF-1α was intended to improve the chondrogenic potential of PB-MSCs by enhancing their recruitment, proliferation, and differentiation into chondrocyte-like cells. After being grafted into mice with osteochondral defects for 12 weeks, cell-laden scaffolds demonstrated to significantly stimulate AC regeneration. In particular, histological investigations, immunohistochemistry and Western blot analysis highlighted that mice receiving PB-MSCs-GAIS grafts exhibited upregulated expression of SOX9 gene and key cartilage ECM proteins such as collagen type II and aggrecan. Beyond the positive regenerative outcomes, we believe that this work is informative about the biomaterial-driven control of the local microenvironment in order to amplify the intrinsic chondrogenic potential of PB-MSCs. The observed upregulation of cartilage markers supports the notion that PB-MSCs can be effectively guided toward a chondrogenic phenotype when appropriate biochemical and structural cues are provided.

Consistent with this concept, two recent studies extended the scaffold-based application of PB-MSCs transitioning from rodent to rabbit models. Mao and collaborators (2025) described a novel injectable hydrogel system made from poly (3-hydroxybutyrate-co-3-hydroxyvalerate-co-3-hydroxyhexanoate, PHBVHHx)-Polyethylene Glycol (PEG) with hyaluronic acid (HA) and kartogenin (KGN), and specifically designed for cartilage repair. This thermosensitive scaffold was used to administer PB-MSCs within the damage site in a rabbit model of cartilage lesion. The cell-laden hydrogel promoted robust cartilage regeneration *in vivo*, with improved matrix deposition and expression of cartilage-specific markers (collagen II), highlighting the feasibility of minimally invasive, cell-based regenerative strategies. Similarly, Su and co-workers (2025) reported the implant of biodegradable PHBVHHx-PEG/collagen hydrogel scaffolds carrying PB-MSCs in a rabbit cartilage defect model. These constructs supported cartilage repair by enhancing matrix synthesis, improving defect filling, and modulating the local inflammatory response, underscoring the synergistic role of biomaterial design and PB-MSC biological activity.

In the authors’ view, these studies collectively highlight that the therapeutic performance of PB-MSCs is dependent on the delivery strategy, and that rational biomaterial design might be a key aspect of future clinical success.

Alternatively, regenerative effects derived by scaffold-free therapy were reported by Lin and colleagues (2022), which worked with a murine OA model induced by destabilization of the medial meniscus. In this study, circulating PB-MSCs were locally administered via intra-articular injection, resulting in a significant reduction of cartilage degeneration compared with untreated OA controls. Histological analyses demonstrated improved cartilage structure and proteoglycan preservation, together with decreased expression of catabolic and inflammatory markers. In our opinion, these findings reinforce the concept that PB-MSC treatment can contribute to the modulation of the joint inflammatory milieu and to the restoration of chondrocyte homeostasis, supporting their role in exerting both regenerative and immunomodulatory effects within the osteoarthritic joint.

Remarkably, the regenerative potential of PB-MSCs has also been validated in large-animal cartilage defect models, which are particularly relevant for clinical translation. Zhao et al. (2018) evaluated mobilized PB-MSCs combined with a composite scaffold consisting of DBM carrying BMP-2 and TGF-β3 chitosan sustained-release microspheres and implanted into a pig cartilage defect model. The PB-MSC-DBM constructs significantly enhanced cartilage regeneration compared with controls (i.e., untreated defect group, and scaffold without PB-MSC group), as evidenced by improved macroscopic morphology, histological organization, and integration with surrounding native cartilage. Overall, these preclinical data further support PB-MSC eligibility for osteochondral regeneration therapy in translationally relevant settings.

Intriguing results were also obtained by Zhang and collaborators (2023), who tested the combined therapy based on the administration of PB-MSCs and Platelet-rich plasma (PRP) to rabbit osteoarthritis (OA) models. After surgical induction of OA in the rabbit knee joints to mimic the condition in human patients, the different experimental groups received a) only PB-MSC injections, b) only PRP injections or c) the combination of PB-MSCs and PRP. Rabbits were treated with each therapy up to 12 weeks, then histological and biomolecular analyses were performed to assess the restoration of the osteochondral defects. Experimental evidence highlighted that rabbits receiving the combined therapy (PB-MSCs + PRP) had the most significant AC regeneration, presenting thicker, more intact, and generally healthier cartilage tissue than the other experimental groups. Additionally, the combined therapy significantly decreased synovial inflammation, with significant reduced expression of pro-inflammatory cytokines and preservation of key structural proteins like collagen and aggrecan in AC tissue. Being these proteins crucial for cartilage integrity and function, their preservation supported the efficacy of the combined therapy in regenerating the joint damage (Zhang et al., 2023). Overall, the preclinical outcomes from this research pointed out that the treatment of the OA joint with a combination of PB-MSCs and PRP not only stimulated cartilage regeneration but also reduced inflammation and preserved chondrocytes from further injury. In the perspective of clinical translation, this therapy holds potential to promote cartilage repair and slow the progression of OA, possibly minimizing the need for more invasive approaches like total joint replacement.

Recently, Brondeel and co-workers (2023) developed a veterinary clinical trial to test a less invasive administration route of xenogeneic equine PB-MSCs via intravenous (IV) injection in dogs suffering from OA, marked by joint degeneration, chronic articular pain and lameness. The 35 dogs enrolled by the study received a single IV dose of PB-MSCs and were monitored for possible side effects and complications, as well as the overall safety of the therapy. Efficacy investigations were performed by objective measures, such as gait analysis, and subjective evaluations, including owner-reported pain and mobility scores, over two defined follow-up periods (i.e., 3- and 6-week post-treatment). Collected data indicated that the IV infusion of xenogeneic PB-MSCs was well-tolerated by the subjects, with no significant adverse reactions to the cell graft. Additionally, preliminary efficacy outcomes reported reduction in pain and increased mobility in several subjects. This study provides initial evidence supporting the safety and effectiveness of intravenous MSC administration in OA treatment, validating the use of xenogeneic stem cell graft in veterinary medicine.

## 4 Immunomodulatory potential of PB-MSCs

One of the hallmark features of MSCs is their immunomodulatory properties, which make them attractive candidates for managing inflammatory conditions. For example, MSCs have been shown to modulate immune responses by secreting anti-inflammatory cytokines such as IL-10 and TGF-β. MSCs also demonstrated the ability to regulate both innate and adaptive immune cells, including macrophages, neutrophils, natural killer cells, T and B lymphocytes. Their activity seems to be mainly mediated by the secretion of immunomodulatory molecules, like prostaglandin E2 (PGE2), nitric oxide, and indoleamine 2,3-dioxygenase (IDO). Additionally, MSCs exhibit the expression of surface markers (i.e., FasL, PD-L1, and PD-L2) which contribute to their immunosuppressive functions (Müller et al., 2021; Planat-Benard et al., 2021; Jiang and Xu, 2020).

Mesenchymal stem cells isolated from bone marrow, adipose tissue, and dental tissues have been largely explored for their capacity in modulating immune responses, whereas the existing literature does not report specific research about the immunomodulatory properties of PB-derived MSCs.

Notably, the phase I/II clinical trial by Zhao and colleagues (2013) tested Stem Cell Educator Therapy using cord blood-derived multipotent stem cells (CB-MSCs) to target insulin resistance in subjects suffering from type 2 diabetes. Patient peripheral blood mononuclear cells were exposed to CB-MSCs to induce immune modulation before reinfusion. The therapy led to improved glycemic control, increased insulin sensitivity, and reduced autoimmune responses. These findings suggest that CB-MSCs can reprogram immune dysfunction, supporting the potential use of blood-derived MSCs in anti-inflammatory and immunomodulatory therapies.

In our opinion, given the well-established role of MSCs in immune-related conditions, there is a compelling need for further research specifically focused on the immunomodulatory properties of peripheral blood-derived MSCs. Advancing our understanding in this area could significantly strengthen the rationale for their integration into clinical applications targeting inflammatory and degenerative diseases like OA.

### 4.1 The immunomodulatory role of PB-MSCs in osteoarthritic cartilage repair

In the context of OA pathophysiology, the immunomodulatory properties of PB-MSCs may hold particular therapeutic relevance. As already outlined, OA is now recognized not only as a degenerative joint disease but also as a chronic inflammatory condition, characterized by synovial membrane activation, recruitment of immune cells (e.g., macrophages, T cells), and sustained overexpression of pro-inflammatory cytokines such as IL-1β, TNF-α, and IL-6. These inflammatory mediators drive cartilage matrix degradation, inhibit chondrocyte anabolism, and contribute to nociceptive signaling and joint pain.

Besides their chondrogenic potential which supports AC regeneration, MSC ability to control immune responses and inflammation could offer novel immunomodulatory therapy to lower pain, slow disease progression and develop more effective, personalized treatments for this debilitating condition.

PB-MSCs exert robust immunomodulatory effects through both cell-cell interactions and paracrine signaling. Mechanistically, PB-MSCs secrete anti-inflammatory cytokines (e.g., IL-10, TGF-β1), prostaglandin E2 (PGE2), nitric oxide (NO), and indoleamine 2,3-dioxygenase (IDO), which suppress T cell proliferation, promote M2 macrophage polarization, and inhibit dendritic cell maturation. Additionally, PB-MSCs express ligands such as PD-L1 and FasL, further supporting immune regulation within the OA joint microenvironment (Müller et al., 2021; Longhini et al., 2019; Ma et al., 2014). These actions not only reduce local inflammation but may also preserve residual cartilage tissue and limit osteophyte formation.

Based on the above considerations, we contend that PB-MSCs possess the dual capability of promoting cartilage regeneration and modulating inflammatory responses, thereby positioning them as particularly compelling candidates for OA therapy. This view is supported by the preclinical study conducted by Wang and colleagues (2024), which demonstrated that intra-articular administration of PB-MSCs in OA models not only improved joint histology and restored ECM composition, but also significantly reduced inflammatory cell infiltration, highlighting their combined regenerative and immunoregulatory potential.

In future clinical trials, PB-MSC-based approaches may be integrated into combinatorial strategies involving biologically active agents such as hyaluronic acid (HA), platelet-rich plasma (PRP), or bioengineered scaffolds to synergistically enhance cartilage repair and modulate joint inflammation. Hyaluronic acid, commonly used for viscosupplementation, can serve as a supportive carrier matrix for PB-MSCs, improving their retention and viability in the synovial environment.

Platelet-rich plasma, rich in growth factors like PDGF, TGF-β, and IGF-1, may further amplify the regenerative and immunomodulatory effects of PB-MSCs by stimulating their proliferation, differentiation, and paracrine activity.

Additionally, the use of bioengineered scaffolds incorporating anti-inflammatory cytokines, extracellular matrix components, or slow-release nanoparticles can create a more favorable microenvironment for tissue integration and long-term repair.

From our perspective, such multimodal interventions may simultaneously target structural regeneration and immune dysregulation, representing a promising innovation in OA therapy. We believe these integrative strategies have the potential to shift clinical paradigms from symptomatic management toward true disease modification. Furthermore, they align well with the principles of personalized medicine, enabling patient-specific therapeutic tailoring based on molecular and pathological profiles. In our opinion, combination therapies based on PB-MSCs might become pivotal in the development of mechanism-driven, durable treatments aimed at halting or reversing OA progression while restoring joint function and improving long-term outcomes.

## 5 Discussion

Despite the promising potential of PB-MSCs in regenerative therapies like cartilage restoration in OA, several challenges remain to be addressed before their potential can be fully realized in the clinical setting. As already discussed, one of the main key issues is the low yield and heterogeneity of stem cells isolated from peripheral blood. Peripheral blood contains relatively low number of MSCs in comparison with other sources, so advanced enrichment techniques are needed to increase cell yield. To overcome this constraint, the optimization of mobilization and enrichment strategies -such as granulocyte colony-stimulating factor (G-CSF) priming and apheresis - could enable more consistent and reproducible cell recovery (Yosupov et al., 2017).

Regulatory hurdles might also represent a significant limit to the clinical translation of PB-MSCs for OA treatment. In the perspective of their grafting into OA human subjects, PB-MSCs must undergo rigorous characterization and meet strict regulatory standards, which could delay their translation from the laboratory to patient care. In this context, the establishment of standardized and GMP-compliant procedures for PB-MSC isolation, expansion, and cryopreservation is particularly relevant, as these steps are essential to ensure product quality, safety, and inter-laboratory reproducibility. In parallel, the development of robust potency assays capable of reflecting both the chondrogenic differentiation capacity and the immunomodulatory activity of PB-MSCs is urgently required to support reliable assessment of therapeutic efficacy.

Careful patient selection is of paramount importance for clinical translation. PB-MSCs represent a suitable treatment option for patients with focal articular cartilage defects or suffering from early/moderate OA, who are not yet eligible for joint replacement. Another clinical indication involves cases when factors like age, comorbidities, or previous surgeries at the donor site can affect candidacy for bone marrow harvest. In addition, the minimally invasive method for PB-MSC collection supports their use in patients requiring repeated cell administrations or preferring lower donor site morbidity. Conversely, PB-MSCs should not be considered a therapeutic approach for subjects diagnosed with hematological cancers (e.g., leukemia, lymphoma), as circulating stem cell subsets may be affected by malignant cells. Systemic infections and bone marrow suppression syndromes can also be included among contraindications for PB-MSC administration. Notably, subjects with bleeding disorders or receiving anticoagulant therapy should be thoroughly assessed before being subjected to the venipuncture procedure. Overall, defining patient eligibility based on well-defined parameters represents one of the key prerequisites for regulatory approval of PB-MSC therapies and their translation into the clinical setting.

Another aspect worth considering is the integration of PB-MSCs with advanced biomaterials, extracellular-vesicle derivatives, and mechano-responsive scaffolds may further potentiate their regenerative and immunomodulatory potential, ultimately enhancing therapeutic efficacy and durability (Tsiapalis and O’Driscoll, 2020; Cosson et al., 2015).

Finally, *in vivo* investigations of PB-MSC immunomodulatory properties and preclinical research on OA animal models should also be improved to get novel insights about the therapeutic potential of these cells. This would lead to more consistent and reproducible results, improving the reliability of PB-MSC grafts for OA clinical treatment.

In conclusion, peripheral blood-derived MSCs are turning out to be a cutting-edge frontier in regenerative medicine and immunotherapy. Their accessibility, autologous origin and multi-differentiation capacity make them a viable alternative to traditional stem cell sources. Recent advancements in isolation techniques, culture systems, and preclinical applications underscore their potential to support OA treatment by stimulating cartilage regeneration and slowing down joint degeneration.

From a regulatory and ethical standpoint, the creation of optimized approval frameworks and dedicated PB-MSC biobanks could significantly facilitate early-phase clinical translation, accelerating the transition from preclinical validation to human application. In our view, PB-MSCs have the potential to redefine the paradigm of autologous regenerative therapy. Their non-invasive collection allows for longitudinal sampling and individualized monitoring, which are essential features for future precision-regenerative approaches.

Ongoing research and clinical trials are expected to address current limitations of PB-MSC therapy and unlock novel applications, making them key elements of next-generation medical care, also in the context of OA management. Continuing to explore the potential of PB-MSCs could open novel opportunities for regenerative therapies and personalized medicine, modulating treatments on the individual patient profile.

As a perspective article, this work presents some limitations that should be acknowledged. Differently from systematic reviews or meta-analyses, this article does not rely on standardized search strategies, well-established inclusion/exclusion criteria, or quantitative data synthesis. This work discusses some literature which was selected to illustrate the main current findings and emerging trends rather than to provide a comprehensive overview of available evidence on the topic. For this reason, the risk of selection bias cannot be completely excluded, and the derived considerations should be interpreted as expert opinion intended to promote discussion and highlight future research perspectives, rather than as definitive evidence-based recommendations.

## Data Availability

The original contributions presented in the study are included in the article/supplementary material, further inquiries can be directed to the corresponding author.
